# Psychotropic drug use among children and adolescents in the Nordic countries - A systematic literature review

**DOI:** 10.1192/j.eurpsy.2022.1080

**Published:** 2022-09-01

**Authors:** D. Ollerup, S. Elkrog, M. Stoltz-Andersen, H. Stubmark, L. Rasmussen, R. Wesselhoeft

**Affiliations:** University of Southern Denmark, Clinical Pharmacology, Pharmacy And Environmental Medicine, Department Of Public Health, Odense, Denmark

**Keywords:** Nordic countries, systematic review, children and adolescents, psychotropics drug use

## Abstract

**Introduction:**

The Nordic countries have similar health care and welfare systems, and rather homogenous populations. Therefore, it is reasonable to expect similar use of psychotropic drugs. However, recent studies show marked differences in a range of psychotropic drug classes among children and adolescents in Sweden, Norway, and Denmark.

**Objectives:**

To review the literature regarding psychotropic drug use among children and adolescents in the Nordic countries.

**Methods:**

We performed a critical systematic literature review according to PRISMA guidelines and registered the study protocol at PROSPERO. Three scientific databases were used: PsycINFO, EMBASE and PubMed. Inclusion criteria were: 1) Age: 3-19 years, 2) Country: Denmark, including Faroe Islands and Greenland, Sweden, Norway, Finland, including Aland, and Iceland, 3) Drug of interest: Psychotropics, including Antidepressants, ADHD medication, antipsychotics, hypnotics, anxiolytics, 4) Population based study sample, 5) Observational study design, 6) Original data, 7) English language, 8) Publication date: 2010-2021. The review process was performed by four reviewers in three steps: 1) title/abstract screening, 2) full text screening, and 3) data extraction, including risk of bias assessment. Before study initiation, acceptable interrater reliability was ensured by pilot tests.

**Results:**

The literature search was conducted October 6^th^, 2021. The PsycINFO database gave 285 hits, EMBASE 1190 hits and PubMed 2185 hits. In total, the literature search gave 3660 hits, of which 294 were duplicates, leaving us with 3366 references. The first screening phase is in process and results will be presented at the EPA conference.

**Conclusions:**

The results of the systematic review will be interpreted and discussed.

**Disclosure:**

No significant relationships.

